# Increased Functional Brain Network Efficiency During Audiovisual Temporal Asynchrony Integration Task in Aging

**DOI:** 10.3389/fnagi.2018.00316

**Published:** 2018-10-09

**Authors:** Bin Wang, Peizhen Li, Dandan Li, Yan Niu, Ting Yan, Ting Li, Rui Cao, Pengfei Yan, Yuxiang Guo, Weiping Yang, Yanna Ren, Xinrui Li, Fusheng Wang, Tianyi Yan, Jinglong Wu, Hui Zhang, Jie Xiang

**Affiliations:** ^1^College of Computer Science and Technology, Taiyuan University of Technology, Taiyuan, China; ^2^Department of Radiology, First Hospital of Shanxi Medical University, Taiyuan, China; ^3^Translational Medicine Research Center, Shanxi Medical University, Taiyuan, China; ^4^Department of Psychology, Faculty of Education, Hubei University, Wuhan, China; ^5^Medical Humanities College, Guiyang University of Traditional Chinese Medicine, Guiyang, China; ^6^Suzhou North America High School, Suzhou, China; ^7^Hefei No. 8 Senior High School, Hefei, China; ^8^School of Life Science, Beijing Institute of Technology, Beijing, China; ^9^Key Laboratory of Convergence Medical Engineering System and Healthcare Technology, Ministry of Industry and Information Technology, Beijing Institute of Technology, Beijing, China; ^10^Key Laboratory of Biomimetic Robots and Systems, Ministry of Education, Beijing Institute of Technology, Beijing, China; ^11^Graduate School of Natural Science and Technology, Okayama University, Okayama, Japan

**Keywords:** functional connectivity, EEG, audiovisual integration, aging, theta and alpha bands

## Abstract

Audiovisual integration significantly changes over the lifespan, but age-related functional connectivity in audiovisual temporal asynchrony integration tasks remains underexplored. In the present study, electroencephalograms (EEGs) of 27 young adults (22–25 years) and 25 old adults (61–76 years) were recorded during an audiovisual temporal asynchrony integration task with seven conditions [auditory (A), visual (V), AV, A50V, A100V, V50A and V100A]. We calculated the phase lag index (PLI)-weighted connectivity networks modulated by the audiovisual tasks and found that the PLI connections showed obvious dynamic changes after stimulus onset. In the theta (4–7 Hz) and alpha (8–13 Hz) bands, the AV and V50A conditions induced stronger functional connections and higher global and local efficiencies, reflecting a stronger audiovisual integration effect, which was attributed to the auditory information arriving at the primary auditory cortex earlier than the visual information reaching the primary visual cortex. Importantly, the functional connectivity and network efficiencies of old adults revealed higher global and local efficiencies and higher degree in both the theta and alpha bands. These larger network efficiencies indicated that old adults might experience more difficulties in attention and cognitive control during the audiovisual integration task with temporal asynchrony than young adults. There were significant associations between network efficiencies and peak time of integration only in young adults. We propose that an audiovisual task with multiple conditions might arouse the appropriate attention in young adults but would lead to a ceiling effect in old adults. Our findings provide new insights into the network topography of old adults during audiovisual integration and highlight higher functional connectivity and network efficiencies due to greater cognitive demand.

## Introduction

Humans use various modalities of sensory information (e.g., visual information, auditory information, olfactory information, and tactile information) in everyday life to perceive the outside world. Stimuli from multiple sensory channels are integrated into common perceptual states, which is a process known as multisensory integration (Ernst and Bülthoff, 2004; Senkowski et al., 2008). Among multisensory integration modalities, the most typical is audiovisual integration in humans and animals, which has been represented in several previous studies (Molholm et al., 2002, 2006; Roach et al., 2006; Doehrmann and Naumer, 2008). Recently, our event-related potential (ERP) studies have indicated that audiovisual integration is regulated by temporal factors (Yang et al., 2014; Ren et al., 2017b). The temporal structure of auditory and visual stimuli can intervene in human audiovisual processing. One example from among life experiences is that we generally see lightning and then hear the thunderclap, although the lightning and thunderclap occur simultaneously. This phenomenon indicates that the integration of multiple forms of sensory information obeys the temporal principle (Stein, 2012; Ren et al., 2017a). When auditory and visual signals occur at the same time and location, they tend to be integrated, and temporal proximity is always a necessary factor for the occurrence of audiovisual integration (Van der Burg et al., 2008; Ren et al., 2017b). To allow precise perception of the environment, a temporal window appears in the human brain. When auditory and visual signals fall within this temporal window, the brain will integrate the stimuli. A previous study showed that when the time between the occurrence of auditory and visual stimuli is less than 100 ms, there will be a strong multisensory integration effect (Ren et al., 2017b).

Growing evidence has demonstrated that audiovisual integration can be influenced by development, aging, attention, training and listening experience (Lippert et al., 2007; McNorgan and Booth, 2015; Paraskevopoulos et al., 2015; Yu et al., 2017). For example, Koelewijn et al. (2010) focused on the question of whether multisensory integration is an automatic process, suggesting that multisensory integration is accompanied by attentional processes and that the two can interact in multiple areas of the brain. Moreover, multisensory integration is related to attention and can be modulated by attention; bottom-up mechanisms induced by cross-modal interactions can automatically capture attention to multisensory events (Koelewijn et al., 2010; Talsma et al., 2010). Old adults have been demonstrated to have abnormalities in audiovisual integration (Laurienti et al., 2006; Peiffer et al., 2007). For example, the presentation of audiovisual stimuli accelerated response times in both old and young adults, with a significantly greater gain in old adults (Laurienti et al., 2006; Tye-Murray et al., 2010; Wu et al., 2012). More recently, abnormal audiovisual integration in old adults was also represented by the delayed integration time window and peak time measured by race model analysis, which also reflected a greater effect of audiovisual integration in old adults (Wu et al., 2012; Ren et al., 2017b). It is well known that old adults experience deficits in visual and auditory abilities, such as a decrease in visual acuity (Bäckman et al., 2006; Liu and Yan, 2007) and an increase in auditory threshold (hearing loss). The greater effect of audiovisual integration in old adults has been explained as an effective compensatory strategy to overcome visual and auditory deficits (Peiffer et al., 2007).

Many studies have shown that neural oscillatory responses in the theta, alpha, beta and gamma bands are involved in sensory processing (Yordanova et al., 1998; Sakowitz et al., 2005; Donner and Siegel, 2011). Previous studies have demonstrated that these frequency bands play a role in multisensory attention tasks with different stages and locations (Sakowitz et al., 2005; Friese et al., 2016). Particularly, both theta and alpha bands are organized in fronto-centro-parietal sites (Sakowitz et al., 2005). We found that multisensory attention led to decreased lower-frequency theta and alpha activity in early sensory cortex areas and to increased low-frequency phase synchronization in the frontal cortex (Friese et al., 2016). The theta band appears to be predominantly implicated in cognitive control and short-term memory in audiovisual integration (Demiralp and Başar, 1992; Sakowitz et al., 2000; Keller et al., 2017), while the alpha band seems to be mainly related to the maintenance of sensory information, cognitive control and the suppression of distractions (Masahiro et al., 2010; Başar, 2012). Recently, van Driel et al. (2014) determined the functional role of the coupling of alpha phase dynamics between sensory cortices in integrating cross-modal information over time. Furthermore, previous studies have reported that healthy aging people show lower spontaneous electroencephalogram (EEG) amplitude in the theta and alpha bands than young people (Cummins and Finnigan, 2007; Gaál et al., 2010). Cummins and Finnigan (2007) found a significant decrease in old adults’ mean theta power of resting EEG. Spontaneous EEG measurements from normal aging have demonstrated significantly reduced alpha power at each electrode (Yordanova et al., 1998; Kolev et al., 2002). We speculate that there are significant age differences in audiovisual integration in the low-frequency (theta and alpha) bands.

Audiovisual integration is involved in multiple brain regions, including the occipital, parietal, temporal, and frontal regions (Shams et al., 2005). Previous functional magnetic resonance imaging (fMRI) and EEG studies have yielded evidence of audiovisual integration effect occurrence in regions traditionally considered sensory specific (e.g., primary visual cortex) (Calvert et al., 2000; Macaluso, 2006; Chan et al., 2017; Ren et al., 2017a). Moreover, direct anatomical connections between the occipital regions (visual processing) and superior temporal regions (auditory processing) have confirmed that these regions may play key roles in audiovisual integration (Falchier et al., 2002). These brain regions synergistically complete audiovisual integration. A central question in audiovisual integration is how perceptive functions depend on the integration and coordination of widely distributed brain regions. To answer this question, a key concept is functional connectivity, which involves temporal correlations or synchronization in physiological signals recorded from different brain regions (Lee et al., 2003; Fingelkurts et al., 2005; Stam et al., 2009; Bhattacharya et al., 2018; Wang et al., 2018). Recent studies have shown that the functional connectivity of the brain network is organized in a highly efficient manner, which implies a high level of local efficiency combined with global efficiency of information transfer (Stam, 2004; Bullmore and Sporns, 2009; Heuvel et al., 2009). Numerous studies have applied graph theoretical analysis to evaluate functional connectivity in EEG and MEG data (Smit et al., 2008; Stam et al., 2008; Vecchio et al., 2014). Our previous research first combined the phase synchrony of neuronal oscillations and graph theoretical analysis of network topography to investigate age-related audiovisual integration in EEG data and found that old adults had stronger functional connectivity and higher brain network efficiencies to execute synchronous audiovisual integration in the beta band (Wang et al., 2017). However, it is unclear how changes in functional connectivity and network efficiencies during audiovisual integration tasks with temporal asynchronous stimuli occur in aging.

To clarify the changes in functional connectivity and brain network efficiencies during audiovisual temporal asynchrony integration tasks in old adults, we designed an auditory or visual stimuli discrimination task consisting of three types of stimuli: unimodal visual (V), unimodal auditory (A) and bimodal audiovisual (AV) stimuli (in synchrony and asynchrony). Two subject groups participated in our experiment, and we recorded EEG signals from different brain regions for all tasks. The phase lag index (PLI), a synchronization measure, was used to construct the brain network in the theta, alpha, beta, and gamma frequency bands. We further used graph theoretical analysis to investigate functional connectivity and network efficiency during these audiovisual conditions.

## Materials and Methods

### Participants

Twenty-five old individuals [61–76 years, mean age ± standard deviation (SD), 68.8 ± 0.90] and 27 young individuals (21–25 years, mean age ± SD, 23.1 ± 0.19) participated in this study. All subjects, who were naive to the purpose of the experiment, had normal hearing and normal or corrected-to-normal vision capabilities. All subjects had no history of cognitive impairment and had normal Mini-Mental State Examination (MMSE) scores (>24) adjusted for age and education (Bravo and Hébert, 1997). Moreover, all subjects provided written informed consent for this experiment, which was approved by the ethics committee of Okayama University.

### Stimuli and Task

**FIGURE 1:**
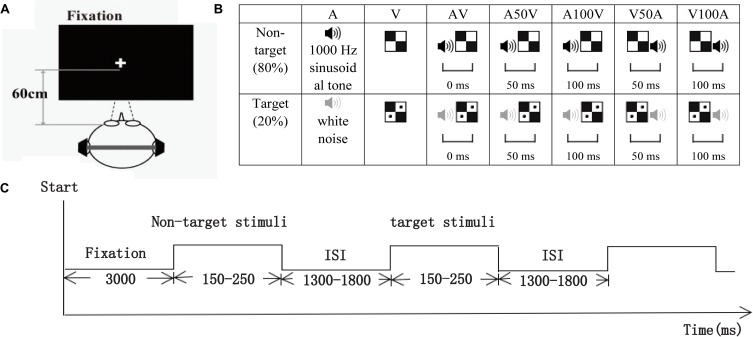
Schematic of the experimental design. **(A)** The subject was approximately 60 cm away from the computer screen used to present visual stimuli. **(B)** The visual non-target stimulus was a black and white checkerboard image, and the visual target stimulus was a black and white checkerboard image containing two black dots. The auditory non-target stimulus was a 1000 Hz sinusoidal tone (black speaker), and the auditory target stimulus was white noise (gray speaker). The audiovisual stimuli included visual and auditory target or non-target stimuli. AV, simultaneous auditory and visual; V50A and V100A, auditory lag visual 50 ms and 100 ms, respectively; A50V and A100V, auditory leading visual 50 and 100 ms, respectively. **(C)** The timeline of one session. Each session started with a 3000 ms fixation period, and then visual, auditory, and audiovisual target or non-target stimuli with a duration of 150–250 ms were randomly presented. All stimuli were presented with an ISI that varied from 1300 to 1800 ms.

The visual non-target stimulus was a black and white checkerboard image, and the visual target stimulus was a black and white checkerboard image containing two black dots (52 mm × 52 mm, with a visual angle of 5°). The auditory non-target stimulus was a 1000 Hz sinusoidal tone, and the auditory target stimulus was white noise. The visual stimuli (V) were randomly presented to the lower left or lower right quadrant of the black screen on a 21-inch computer monitor located 60 cm in front of the participant’s eyes for 150 ms (**Figure 1A**). The auditory stimuli (A) were randomly presented to the left or right ear through earphones at 60 dB SPL for 150 ms. The audiovisual stimuli (AV) were presented in the following conditions, which were a combination of the visual and auditory stimuli with different periods of stimulus onset asynchrony (SOA) of 0 ms, ± 50 ms, or ± 100 ms: simultaneous AV; auditory lag visual 50 ms, 100 ms (V50A, V100A); or auditory leading visual 50 ms, 100 ms (A50V, A100V; **Figure 1B**). Moreover, the non-target stimuli comprised 80% of the total stimuli, and the duration of each trial of each stimulus was between 150 and 250 ms, depending on the SOA that was chosen according to previous behavioral studies (Bolognini et al., 2005; Yang et al., 2014).

All participants were instructed to perform an auditory or visual stimuli discrimination task consisting of visual (V), auditory (A), and audiovisual (AV) stimuli (in synchrony and asynchrony) in this experiment. Eight sessions were conducted for each participant, and each session started with a 3000 ms fixation period and then randomly presented 25 visual stimuli, 25 auditory stimuli and 125 audiovisual stimuli (AV, A50V, A100V, V50A, and V100A). In total, there were 80 non-target stimuli and 20 target stimuli for each condition on the left or right side. All the stimuli were presented with an ISI that varied from 1300 to 1800 ms (**Figure 1C**). The participants were instructed to determine as quickly and accurately as possible whether the targets appeared on the left or right side.

### EEG Data Collection

The EEG was recorded from 32 scalp electrodes mounted within an electrode cap (Easy cap, Germany), which were placed according to the International 10–20 electrode placement standards, and 2 electrooculogram electrodes that were referenced to the earlobes. The EEG signals were amplified by BrainAmp amplifiers (Brain Products, Munich, Germany) using a 0.05 to 100 Hz bandpass and were digitized at a sampling rate of 500 Hz.

### Behavioral Performance and Race Model Analysis

Behavioral performance analyses were performed on the trials with target stimuli. The hit rate (HR) was the correct responses to target stimuli divided by the total number of target stimuli, and the false alarm (FA) rate was the responses to non-target stimuli divided by the total number of non-target stimuli. No subject had behavioral performance with HRs below 70% and FA rates above 30%. The use of cumulative distribution functions (CDFs) to evaluate the race model ensures that multisensory enhancement is identified using the entire response time distribution rather than a single central tendency score (i.e., the mean response time) (Miller, 1982, 1986). To complete this analysis, the CDFs of each condition were performed on the response times by using 10 ms time bins. Unimodal CDFs were used to calculate the race distribution at each time bin with the following formula: [P (A) + P (V)] - [P (A) ^∗^ P (V)]. Each participant’s race model curve was then subtracted from their audiovisual CDFs. The peak time point of each probability difference curve was recorded and represented the time at which the audiovisual integration most likely occurred. For more details, please refer to our previous behavioral study with a similar design (Ren et al., 2017b).

### Preprocessing of EEG Data

The EEG data were preprocessed using Brain Vision Analyzer software (version 2.02, Brain Products GmbH, Munich, Bavaria, Germany) and MATLAB R2014a (MathWorks Inc., Natick, MA, United States) with the following open-source toolboxes: EEGLAB^1^ (Swartz Center for Computational Neuroscience, La Jolla, CA, United States). Brain Vision Analyzer software was used to automatically reject trials with electrocardiographic activity or eye and muscle artifacts from the data for all electrodes. The EEG data were divided into epochs with 700 time points, which were from 500 ms before the stimulus onset to 900 ms after the stimulus onset, followed by baseline correction for each epoch. In the present study, we analyzed only non-target stimuli to avoid effects of motor responses. Some studies have reported the same results for responses to both non-target and target stimuli (Missonnier et al., 2007; Kukleta et al., 2009). The epochs of non-target stimuli were filtered into theta (4–7 Hz), alpha (8–13 Hz), beta (14–30 Hz), and gamma (31–50 Hz) frequency ranges. The network synchronization of these four bands was investigated, as these rhythms are considered particularly relevant for interregional communication (Lewis, 2005; Sakowitz et al., 2005; Palva and Palva, 2007; Donner and Siegel, 2011).

### Interregional Phase Synchronization

Interregional phase synchronization was evaluated by calculating the PLI and was analyzed by an in-house program in MATLAB. First, the Hilbert transform was employed to obtain time series of instantaneous phase measures for each trial epoch and each electrode. Then, the locked-phase synchrony was indexed by PLI, which measures the asymmetry of the phase difference distribution between two electrodes at a given time point across trials.

$$PLI=|sign\left(\Delta \phi \left(t_{n}\right))|=|\frac{1}{N}\overset{N}{\underset{n=1}{\Sigma }}sign\left(\Delta \phi (t_{n})\right)\right|$$

The PLI analysis produces an electrode-by-electrode adjacency matrix (30 × 30) across trials (700 time points). We did not display the first and last 300 ms (150 sample points) in the synchrony analyses of epochs because of distortions involved in calculating the Hilbert transform at the edges of the analyzed epochs (Doesburg et al., 2008; Leung et al., 2014; Wang et al., 2017; Yan et al., 2017). Thus, we reported only the adjacency matrix for reduced epochs (400 time points) from 200 ms before to 600 ms after stimulus onset. The PLI analyses were performed on each frequency band, each stimulus condition, and each subject.

### Functional Connectivity

The adjacency matrices at each time point were then averaged for each trial condition. The average network connectivity time series was obtained to investigate functional connectivity dynamics. Permutation tests with 10,000 repeats were employed at each time point to compare the differences in the mean PLI values between the two groups. A time point with significant group differences (permutation test, *p* < 0.05, corrected) reflected a difference in the strength of functional connectivity when processing the stimuli. To easily compare differences in group and condition, the time windows with significant group differences were determined across all conditions. To avoid double dipping, we determined the time windows of each subject by the leave-one-out method and obtained individual time windows. The individual time windows were approximately 0–370 ms after the stimulus for the theta band and approximately 40–220 ms for the alpha band. The averaged adjacency matrices within the time windows represented the functional connectivity for each subject and each condition and were further used in analyses of functional connectivity and network topology.

After the functional connectivity was obtained, the mean weight (PLI) of all connectivities in the network was used to characterize the global network strength. The network strength was measured for each condition and subject. To further identify specific functional connectivities that were different between young and old adults, the connectivity matrices were entered as repeated-measures dependent variables into the network-based statistic (NBS) toolbox. The initial univariate *t*-test for between-group comparisons of interregional connectivity was adapted for the data distributions being analyzed to *T* = 3 (*p* < 0.01) in the 30 × 30 adjacency matrix (Zalesky et al., 2012; Leung et al., 2014; Wang et al., 2017). A surrogate statistical approach with 5,000 repeats was used to determine the statistical significance of connectivity components reflecting group differences in network synchronization.

### Analysis of Network Topologies

To characterize the network topologies, we constructed a weighted network from connectivity matrices (30 × 30) for each condition, frequency band, and subject. To compare group differences in topological metrics between brain functional networks regardless of the selection of specific thresholds, we calculated the g topological metrics of the networks with a range of sparsity thresholds from the top 5–40% of individual subject connections. The network metrics, including global efficiency (Eg), local efficiency (Eloc) and degree, were calculated by GRETNA (Wang et al., 2015). Global efficiency (Eg) measures the capacity to integrate information across the network. This metric is the inverse of the average shortest path length from one node to all other nodes (Sporns, 2011; Barbey, 2017; Wig, 2017). Local efficiency (Eloc) measures the local information processing capabilities of the network and reflects the fault tolerance of the network. This metric is the inverse of average shortest path length of the neighbors of a node (Sporns, 2011; Wig, 2017). The degree measures the connectivity of each node (Burdette et al., 2010). Finally, we characterized the integrated metric for each network metric by calculating the area under the curve (AUC) for sparsity thresholds from 5 to 40% in order to provide a scalar that did not depend on the specific threshold selection.

### Statistical Analysis

All statistical analyses were performed using SPSS, version 20.0 (SPSS, Inc., Chicago, IL, United States). For each frequency, a repeated-measures ANOVA was carried out separately for the behavioral performance and network metric. To examine the effects of age and conditions as well as their interaction, a 2 groups (old, young) × 7 conditions (A, V, AV, A50V, A100V, V50A, V100A) repeated-measures ANOVA was performed. For any difference with *p* < 0.05 (Mauchly’s sphericity test), *post hoc* tests were performed by a permutation test (10,000 repeats) for group differences, and a paired-permutation test (10,000 repeats) was performed for condition differences. These multiple comparisons were corrected by the Bonferroni method at the condition level. To examine the relationship between topological properties and integration performance, a mixed-effects model was used to test the correlations between each network metric and peak time. The mixed-effects model reflected only the global relationship across all conditions. To evaluate correlations in each condition in greater detail, Spearman correlations were also used to measure the relationship between topological properties and integration performance.

## Results

### Behavioral Performance

**FIGURE 2:**
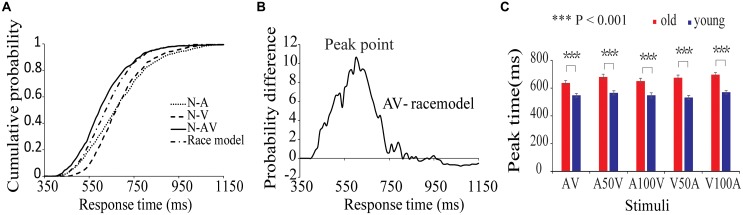
Cumulative probability functions and race model. **(A)** Cumulative distribution functions (CDF) for response times to visual, auditory, and audiovisual stimuli in the two age groups. The summed probability of visual and auditory responses is shown by the race model curve (race model). **(B)** The curve of probability difference between the CDFs and race model. A positive difference indicates the existence of audiovisual integration in the behavioral performance. **(C)** The peak time point is the significant difference between the two groups in each audiovisual condition.

The average HRs of old adults and young adults were 93.7 and 95.2%, respectively. For detailed results of the race model, please refers to our previous behavioral study with a similar design (Ren et al., 2017b). In the present study, the results of CDFs of V, A, AV and the race model of all subjects are shown in **Figure 2A**. The distribution of CDFs revealed that the responses to the AV stimuli were faster than the response to V or A stimuli. Furthermore, to identify whether audiovisual integration occurred, we measured the probability difference by subtracting the CDFs of AV from the race model. **Figure 2B** shows the mean probability difference of all subjects. The peak time point of each probability difference curve reflects behavioral facilitation during audiovisual integration. Thus, the probability differences were calculated for each audiovisual condition and each subject. The peak time points of all audiovisual conditions are shown in **Figure 2C**. The repeated-measures ANOVA revealed that peak time had a significant main effect of group [*F*(1,50) = 26.731, *p* < 0.001]. Further *post hoc* tests showed that old adults had a longer peak time than young adults in all conditions (**Figure 2C**, paired-permutation test, *p* < 0.001, Bonferroni corrected). There were significant main effects of condition [*F*(4,200) = 11.230, *p* < 0.001] and interaction between group and condition [*F*(4,200) = 4.076, *p* = 0.007]. The pairwise comparison showed that the young adults had the shortest peak time in the V50A condition, and the old adults had the shortest peak time in AV (permutation test, *p* < 0.05, corrected).

### Time Courses of Mean PLI

**FIGURE 3:**
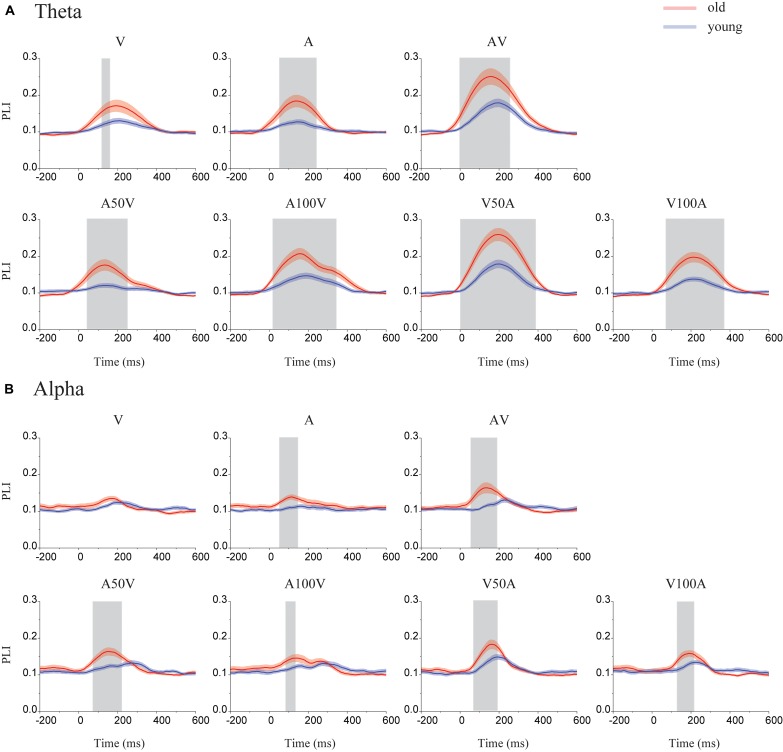
Time courses of the mean PLI in the theta and alpha bands. **(A)** In the theta band, the time courses of the mean PLI for each group in each condition (including the A, V, AV, A50V, A100V, V50A, and V100A conditions). **(B)** In the alpha band, the time courses of the mean PLI for old (red line) and young adults (blue line) included time points of 200 ms before stimulus onset and 600 ms after stimulus onset. The shaded areas indicate the time periods with significant group differences (permutation test, *p* < 0.05, corrected).

The PLI or network metrics of the left and right sides were averaged because there were no differences between them. The time courses of the PLI for old adults and young adults are shown in **Figure 3**. The results showed obvious dynamic changes after stimulus onset for all conditions, especially for the audiovisual conditions. We found differences in PLI between young and old adults in the theta and alpha bands (**Figure 3**) but not in the beta or gamma bands. Significantly higher values of PLI curves were mainly found within the range of 0-370 ms for the theta band (permutation test, *p* < 0.05, corrected) and 40–220 ms for the alpha band (permutation test, *p* < 0.05, corrected).

### Functional Connectivity

**FIGURE 4:**
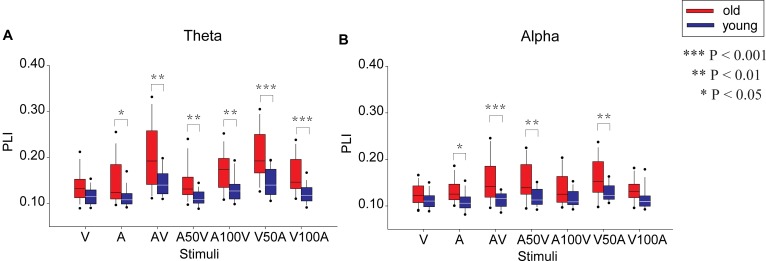
Comparison of the mean PLI between old and young adults in the seven conditions (A, V, AV, A50V, A100V, V50A, and V100A) in the theta **(A)** and alpha **(B)** bands.

The network strengths were measured as the mean weight (value of PLI) of all connectivities (**Figure 4**), which were used to characterize the global weighted network during audiovisual tasks. By repeated-measures ANOVA, the significant main effects of the stimulus condition were determined in the network strengths of the theta [*F*(6,300) = 39.965, *p* < 0.001] and alpha bands [*F*(6,300) = 12.346, *p* < 0.001]. In the theta band, a pairwise comparison showed stronger connectivity strengths in the V50A and AV conditions than in all other conditions (paired-permutation test, *p* < 0.01, corrected). There were stronger connectivity strengths in multimodal stimulation conditions (AV, V50A, A100V, and V100A) than in unimodal stimulation (A or V, paired-permutation test, *p* < 0.05, corrected). For the alpha band, the V50A condition showed the strongest connectivity strengths among all conditions (paired-permutation test, *p* < 0.05, corrected). There were stronger connectivity strengths in the three types of multimodal stimulation conditions (AV, A50V, and V50A) than in the unimodal stimulation (A or V, paired-permutation test, *p* < 0.05, corrected). More details of the pairwise comparisons are shown in **Supplementary Tables S1**, **S2**.

In addition, the network strengths in the theta and alpha bands showed a significant main effect of group {theta: [*F*(1,50) = 17.190, *p* < 0.001], alpha: [*F*(1,50) = 13.139, *p* = 0.001]} and a significant interaction between group and condition [theta: *F*(6,300) = 3.744, *p* = 0.010, alpha: *F*(6,300) = 3.838, *p* = 0.005, **Figure 4**]. For the theta band, the *post hoc* tests showed that old adults had significantly stronger connectivity strengths than young adults in all conditions except the V condition (permutation test, *p* < 0.05, corrected, **Figure 4A**, permutation test), especially in audiovisual conditions (*p* < 0.01, corrected). In the alpha band, old adults had significantly stronger connectivity strengths than young adults in the AV, A50V, and V50A conditions (**Figure 4B**, permutation test, *p* < 0.01, corrected).

**FIGURE 5:**
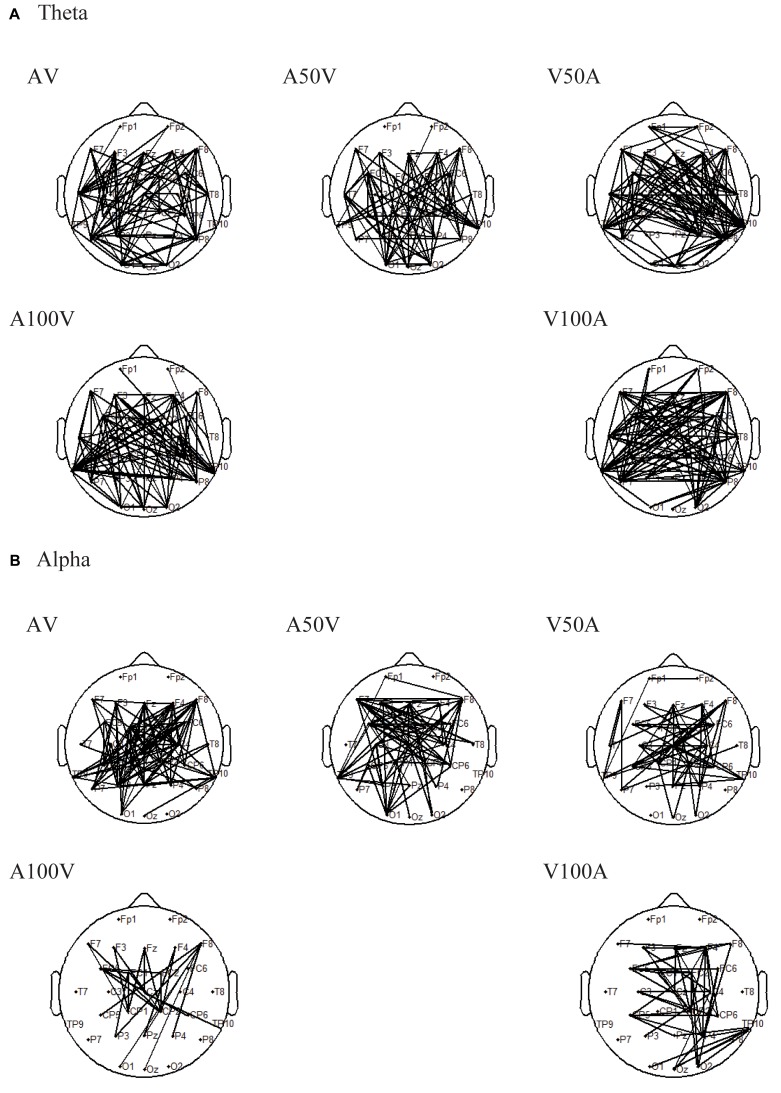
Results of pairwise associations. We used the NBS to identify interregional connectivity that significantly differed between the groups for the theta and alpha bands. **(A)** In the theta band, old adults had significantly stronger connections in all of the audiovisual conditions. **(B)** In the alpha band, old adults had significantly stronger connections in all of the audiovisual conditions.

Connectivity alteration in the functional network is the foundation of a difference in network strength. Thus, we further used an NBS approach to localize the specific functional connectivity that was significantly enhanced in old adults compared with young adults (**Figure 5**). In the theta band, old adults had significantly stronger connections in all of the audiovisual conditions (AV, A50V, V50A, A100V, and V00A, **Figure 5A**, *p* < 0.001, NBS corrected). In addition, in the alpha band, old adults had significantly stronger connectivity in all of the audiovisual conditions, especially in the AV, A50V, and V50A conditions (**Figure 5B**, *p* < 0.001, NBS corrected). The significantly stronger connections were mainly located in the fronto-centro-parietal site and the superior temporal site (**Figure 5**). The specific functional connectivity was significantly altered in old adults, which was attributed to the differences in topological properties between young and old adults.

### Statistical Results of Network Metrics

**FIGURE 6:**
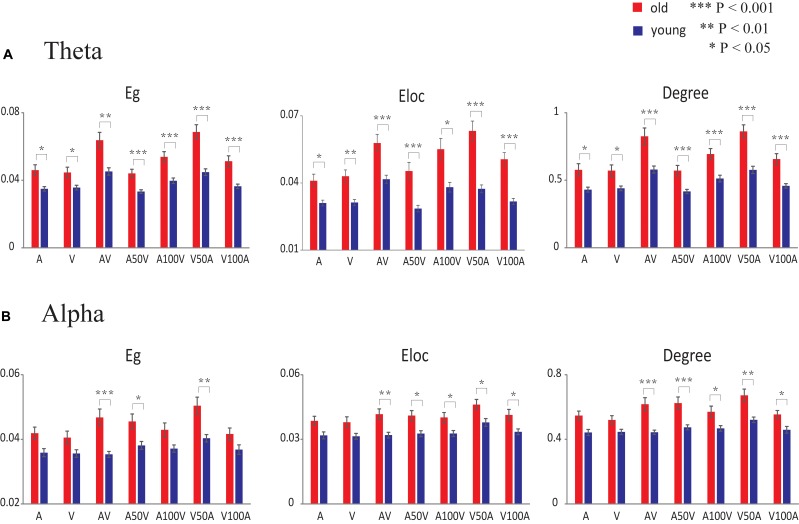
Statistical results of network metrics for each condition. **(A)** In the theta band, the comparison of network metrics between the two groups in each condition. **(B)** In the alpha band, the comparison of network metrics between the two groups in each condition. The error bar indicates the standard error of the mean. Eg, global efficiency; Eloc, local efficiency.

For the theta band, the global efficiency, local efficiency, and degree showed significant main effects of group [Eg: *F*(1,50) = 20.259, *p* < 0.001; Eloc: *F*(1,50) = 23.465, *p* < 0.001; degree: *F*(1,50) = 20.688, *p* < 0.001] and stimulus condition [Eg: *F*(6,300) = 48.302, *p* < 0.001; Eloc: *F*(6,300) = 31.634, *p* < 0.001; degree: *F*(6,300) = 46.827, *p* < 0.001]. There were significant interactions between group and condition [Eg: *F*(6,300) = 6.214, *p* = 0.001; Eloc: *F*(6,300) = 5.385, *p* < 0.001; degree: *F*(6,300) = 4.116, *p* = 0.006]. The pairwise comparison revealed that the AV and V50A conditions were significantly larger than all other conditions (paired-permutation test, *p* < 0.001, corrected). The global and local efficiencies and degree in unimodal stimulation conditions (A or V, paired-permutation test, *p* < 0.001) were smaller than those in multimodal stimulation conditions (AV, V50A, and A100V). More details of the pairwise comparison are shown in **Supplementary Table S1**. Moreover, the *post hoc* tests showed that old adults had higher values of global efficiency, local efficiency and degree than young adults in all conditions (**Figure 6A**, permutation test, *p* < 0.05, corrected). There were more significant group differences in the multimodal conditions, especially in the AV, V50A, and V100A conditions (permutation test, *p* < 0.01, corrected), than in the unimodal conditions.

For the alpha band, the network metrics of global efficiency, local efficiency, and degree showed significant main effects of group [Eg: *F*(1,50) = 11.824, *p* = 0.001; Eloc: *F*(1,50) = 12.812, *p* = 0.001; degree: *F*(1,50) = 15.345, *p* < 0.001] and condition [Eg: *F*(6,300) = 11.836, *p* < 0.001; Eloc: *F*(6,300) = 7.095, *p* < 0.001; degree: *F*(6,300) = 12.495, *p* < 0.001]. Moreover, significant interactions between group and condition were observed only in global efficiency and degree [Eg: *F*(6,300) = 3.321, *p* = 0.010; Eloc: *F*(6,300) = 0.366, *p* = 0.861; degree: *F*(6,300) = 2.872, *p* = 0.025]. A pairwise comparison showed that the higher global and local efficiencies and degree in the V50A condition had the largest value among these conditions (paired-permutation test, *p* < 0.05, corrected). In addition, the A50V condition had a significantly higher global efficiency and degree than the A and V conditions (paired-permutation test, *p* < 0.05, corrected). More details of the pairwise comparison are shown in **Supplementary Table S2**. Compared with young adults, the old adults had significantly higher global efficiency in the AV, A50V, and V50A conditions and higher local efficiency and degree in all audiovisual conditions, especially in the AV, A50V, and V50A conditions (**Figure 6B**, permutation test, *p* < 0.05, corrected).

### Relationship Between Network Metrics and Integration Performance

**FIGURE 7:**
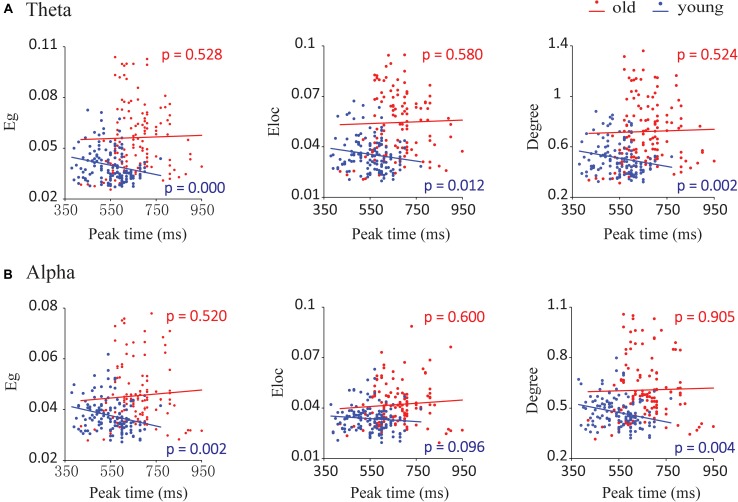
Correlations between network parameters and the peak time point of cumulative probability difference curves. **(A)** In the theta band, global efficiency (Eg), local efficiency (Eloc), and degree had significant negative correlations with the peak time for young adults during audiovisual stimuli. **(B)** In the alpha band, we detected that global efficiency and degree had significant negative correlations with the peak time for young adults during audiovisual stimuli.

There were significant associations between network metrics and the peak time of the audiovisual conditions in young adults but not in old adults (**Figure 7A**). By mixed-effects model analysis, only young adults showed significant negative correlations between peak time and global efficiency (*F* = 13.818, *p* = 0.000), local efficiency (*F* = 6.543, *p* = 0.012) and degree (*F* = 10.500, *p* = 0.002) in the theta band (**Figure 7A**). To further determine the correlation in each condition, we performed Spearman correlations between the network metrics and peak time for each condition and each group. These network metrics in the AV, A50V, and V50A conditions showed significant correlations with peak time (Spearman correlations, *r* > -0.416, *p* < 0.05). Scatterplots for each condition with significant correlations are shown in **Supplementary Figure S1**. For the alpha band, global efficiency and degree had significant negative corrections with peak time in young adults (Eg: *F* = 9.927.818, *p* = 0.002; Eloc: *F* = 2.815, *p* = 0.096; degree: *F* = 8.739, *p* = 0.004, **Figure 7B**). The network metrics in the AV and V50A conditions were also determined to have significant correlations with peak time (Spearman correlations, *r* > -0.397, *p* < 0.05, **Supplementary Figure S2**).

## Discussion

The goal of the present study was to examine whether functional connectivity during an audiovisual temporal asynchrony integration task was influenced by aging. There were significant differences between the two age groups within 200 and 400 ms after stimulus onset in the alpha and theta bands, respectively. The old adults had stronger functional connectivity in the theta band for all audiovisual stimuli (AV, A50V, V50A, A100V, and V100A) and in the alpha band especially for some audiovisual stimuli (AV, A50V, and V50A). This result was confirmed by graph theoretical analysis, which showed that significantly higher global and local efficiencies and degree were found in old adults. In addition, the network efficiencies were significantly associated with the peak time of integration only in young adults. Together, our findings provide new insights into the network topography of old adults during audiovisual integration tasks with temporal asynchronous information.

### Audiovisual Integration and Temporal Synchrony

Research on age-related audiovisual integration has attracted much interest in recent years, as sensory systems and cognitive functions undergo significant changes with age. Brain function is based on the current environment, and different sensory organs are responsible for filtering and screening input information, whereby limited attention is allocated to integrating useful information. In addition, it has been shown that the temporal asynchrony between sensory modalities is a key factor affecting multisensory integration (Durk et al., 2009). To precisely perceive the environment, the human brain defines a temporal window, and when a pair of audiovisual signals fall within this window, the two signals are integrated by the brain (Durk et al., 2009; Ren et al., 2017b). Furthermore, our previous behavioral and ERP studies revealed that integration occurs when the two stimuli fall within a temporal window ranging from 0 to 100 ms (Ren et al., 2017b).

It is worth noting that we found that both old and young adults had significantly higher functional connectivity and network metrics (including global and local efficiencies and degree) under the AV and V50A conditions than under other conditions. Our findings indicated that the strongest audiovisual integration effects occurred in the AV and V50A conditions, which might be due to the auditory information arriving at the primary auditory cortex earlier than the visual information reaching the primary visual cortex (King and Palmer, 1985). Molholm et al. (2002) studied the onset of early audiovisual integration and found that the onset latency of visual stimuli was approximately 50 ms, whereas the onset latency of auditory stimuli was less than half that of visual stimuli (9–15 ms from stimulus presentation). The auditory and visual neural signals arrive at the brain at closer times, resulting in a larger integration effect.

### Increased Phase Synchronization of Neural Oscillations in Aging

The theta and alpha bands play a major role in audiovisual integration (Sakowitz et al., 2005; Başar, 2012; van Driel et al., 2014; Friese et al., 2016; Keller et al., 2017). Both old and young adults showed increased PLIs in the alpha and theta bands after stimulus onset, especially for audiovisual conditions (**Figures 3**, **4**). Researchers have reported evidence that EEG oscillations in the alpha and theta bands particularly reflect attention and cognitive control (Klimesch, 1999; Sakowitz et al., 2005; Friese et al., 2016; Keller et al., 2017). In addition, an EEG revealed increased phase locking in the theta and alpha bands between regions of the human brain in a difficult working memory task (Halgren et al., 2002). We further found age-related differences only in the theta and alpha bands in audiovisual conditions. The significantly higher PLI for old adults during audiovisual conditions indicates the presence of stronger phase locking in old adults while performing tasks. Some studies have examined age-related differences in multisensory integration, demonstrating that old individuals exhibit greater multisensory integration than young individuals (DeLoss et al., 2013; Stevenson et al., 2015). The results of functional imaging studies have also suggested that there is increased activity in the brain network during motor tasks with aging (Seidler et al., 2010; Schmiedtfehr et al., 2016).

The difference in connection revealed by NBS also confirmed the mean PLI of global connections. We found significantly stronger connections, mainly located in the fronto-parietal site and superior temporal site, in old adults than in young adults in the audiovisual conditions only (**Figure 5**). Consistently, van Driel et al. (2014) determined the functional role of the coupling of alpha phase dynamics between sensory cortices in integrating cross-modal information over time. A recent report also found that multisensory attention leads to increased low-frequency phase synchronization in the frontal cortex (Friese et al., 2016). These connection patterns might imply that the theta band plays a role in cognitive control and short-term memory in audiovisual integration (Demiralp and Başar, 1992; Sakowitz et al., 2000; Keller et al., 2017), and the alpha band seems to be mainly related to the maintenance of sensory information, cognitive control and suppression of distractions (Masahiro et al., 2010; Başar, 2012). Thus, a possible explanation could be that the greater connections in the theta and alpha bands are likely due to cognitive demand and attention maintenance.

### Age-Related Alteration in Network Efficiencies of the Theta Band

In the present study, we found a significant increase in theta band functional connectivity, which was mainly located in frontal, parietal, and temporal sites (**Figures 4A**, **5A**), as well as in the network metrics (global and local efficiencies and degree) during audiovisual stimuli in old adults (**Figure 6A**). Previous studies also showed that old adults had greater responses in the theta band during sensory processing (Yordanova et al., 1998; Dushanova and Christov, 2014). Theta activity is generally thought to be associated with a general brain integrative mechanism (Sauseng et al., 2007, 2010) and central executive functions during audiovisual integration (Masahiro et al., 2010). In working memory tasks, interregional theta synchronization has been shown to be related to integration mechanisms (Sauseng et al., 2010; Keller et al., 2017). The topological properties of whole-brain networks support the integrative role of the connection in the theta band. Higher global efficiency of the network corresponds to fast global information communication (Sporns, 2011; Wig, 2017), and local efficiency supports fast local information processing (Sporns, 2011; Wig, 2017). A previous analysis revealed more connectivity, including in the frontal and parietal cortices, during more difficult tasks, demonstrating that audiovisual integration requires not only local information processing within the auditory or visual systems but also global information communication for audiovisual integration and executive control (Sauseng et al., 2007). Recent studies have demonstrated that theta oscillations, mainly located in frontal and parietal regions, are associated with both attention and cognitive control (Friese et al., 2016; Keller et al., 2017). By using the time-window-of-integration model, Diederich et al. (2008) demonstrated that old adults had a smaller probability of integration and larger neural enhancement for neural integration and preparation of an oculomotor response. Moreover, the audiovisual condition showed a larger group difference, especially for the AV, V50A, and V100A conditions, which showed stronger audiovisual integration. Our findings suggest that the higher network efficiency of the theta band in old adults is associated with higher attention and cognitive control and reflects higher cognitive demand in old adults.

### Age-Related Alteration in Network Efficiencies of the Alpha Band

In the alpha band, we found significantly higher functional connectivity, mainly in frontal and parietal sites (**Figures 4B**, **5B**) and in network metrics, in old adults than in young adults in the audiovisual condition (**Figure 6B**). These results indicate that old adults require higher functional connectivity to complete audiovisual integration. Moreover, old adults had significantly higher global and local efficiencies, especially in the AV, A50V, and V50A conditions. There are two potential interpretations of these differences in the alpha band. In general, alpha synchronization reflects an active attentional suppression mechanism or executive functions (Kelly et al., 2006; Masahiro et al., 2010), which are critical for the evocation of a multisensory response (Koelewijn et al., 2010). Diederich et al. (2008) demonstrated that old adults had greater neural enhancement for neural integration and preparation of an oculomotor response. Old adults were more likely to be distracted by irrelevant stimuli and show less efficient inhibitory function than young adults (Andrés et al., 2006; Li et al., 2013). Therefore, alpha phase synchrony was significantly enhanced in old adults during temporal audiovisual integration, supporting the idea that the observed enhancement may function to compensate for distraction and disinhibition in old adults (Li et al., 2013; van Driel et al., 2014). In addition, the alpha amplitudes also involved the maintenance periods in the temporal area for auditory information and the parietal area for visual information (Masahiro et al., 2010). Old adults experience an increase in auditory threshold (hearing loss) and a decrease in visual acuity (Bäckman et al., 2006; Liu and Yan, 2007). These deficits likely result in a smaller probability of integration even with a wider window of integration, produce more cognitive demand and manifest greater neural enhancement (Diederich et al., 2008), which may be attributed to higher functional connectivity and larger network efficiencies.

Furthermore, previous studies on a general audiovisual integration task did not find significant differences between groups in alpha band-related metrics. A possible reason is that under their experimental conditions, which incorporated fewer conditions and less difficulty than our study, old adults did not need greater attentional resources to complete the audiovisual integration task (Wang et al., 2017). Previous studies also demonstrated that the alpha band involves executive functions and the memory storage buffer for auditory and visual information (Masahiro et al., 2010). During the present audiovisual temporal asynchrony integration task, there were more conditions and a higher cognitive demand; therefore, the difference in network efficiencies of the alpha band between young and old adults was likely to become larger.

### Relationship Between Network Metrics and Integration Performance

We found that the time window of behavioral integration in young adults was shorter and less delayed than that in old adults, which is consistent with the findings of our previous behavioral study (Ren et al., 2017b). The peak time point represents the likely occurrence of audiovisual integration. The peak time point of each probability difference curve was recorded for each participant in each group. In the theta and alpha bands, network metrics of audiovisual processing showed strong correlations with peak time points in young adults but not in old adults, especially in the AV, A50V, and V50A conditions. Interestingly, our recent report also demonstrated such correlations in the beta band in old adults but not in young adults (Wang et al., 2017). These findings indicate that the network efficiency reflects the efficient integration of information in the brain. A greater efficiency is associated with a shorter peak time of integration. Moreover, the different design of audiovisual integration of these two reports led to different associations in EEG bands. A previous study demonstrated that activities in the alpha and theta bands appeared as intersensory processing and could therefore participate in different stages of perception and executive functions (Sauseng et al., 2007; Masahiro et al., 2010).

Due to the confusion in temporal asynchrony and more conditions, the audiovisual integration task in the present study was much harder and required more cognitive resources than the standard audiovisual integration task, especially for old adults. The greater cognitive resources required to perform highly demanding tasks would lead to changes in communication within the cortical system (Sauseng et al., 2007). Young adults needed to maintain attention to achieve tasks that lead to associations with integration performance. However, such associations disappeared in old adults because the current task was too hard for them. Moreover, this assumption was also supported by the associations between network efficiency and integration performance only in old adults during a much simpler audiovisual integration task (Wang et al., 2017). There was a ceiling effect in old adults during the audiovisual integration task with temporal asynchrony and a floor effect in young adults during a simple audiovisual integration task.

### Limitations

Our study has several limitations. One limitation is that we chose 32 scalp electrodes to construct the brain network; thus, the number of nodes in the network was relatively small. Second, we performed a PLI analysis and constructed the connection among electrodes. This type of connection has limited ability to reveal the connections among the center regions. However, with EEG source analysis or magnetoencephalography, the topological properties in cortex regions will benefit from investigations of the mechanism of an aging effect during audiovisual integration. Third, we determined the associations between peak time and network metrics only in young adults. The EEG analysis selected trials for non-target stimuli only. The attentional effects between target and non-target stimuli require further investigation, which may clarify the association between integration performance and network metrics. In the future, we will determine how to adjust low-frequency band functional connectivity or temporal asynchrony between inputs to different sensory modalities to improve sensory function, which gradually weakens during normal aging.

## Conclusion

In the present study, for both the theta and alpha bands, the AV and V50A conditions induced stronger functional connections and higher global and local efficiencies, reflecting a stronger audiovisual integration effect, which was attributed to the auditory information arriving at the primary auditory cortex earlier than the visual information reaching the primary visual cortex. The old adults showed a stronger connectivity strength of the theta and alpha bands in the audiovisual temporal asynchrony integration task. The results of the network metrics of old adults also showed higher global efficiency, local efficiency and degree, especially in audiovisual conditions. These larger network efficiencies reflect more demand of attention and cognitive control in old adults, who found the current task more difficult than did young adults. The network efficiencies were significantly associated with the peak time of integration in young adults only, which implied that an audiovisual integration task with multiple conditions might arouse the appropriate attention in young adults but lead to a ceiling effect in old adults. Our findings provide new insights into the network topography of old adults during audiovisual integration tasks with temporal asynchronous information and highlight the higher functional connectivity and network efficiencies due to greater cognitive demand.

## Author Contributions

BW, TianyiY, and JX conceived and designed the experiments. PL, YN, and TL analyzed and interpreted the data, and wrote the paper. DL, RC, PY, YG, TingY, XL, and FW revised the paper. WY, YR, JW, and HZ performed the experiments.

## Conflict of Interest Statement

The authors declare that the research was conducted in the absence of any commercial or financial relationships that could be construed as a potential conflict of interest.

## Footnotes

1 http://sccn.ucsd.edu/eeglab/
