# Supplementary material for: Increased Functional Brain Network Efficiency During Audiovisual Temporal Asynchrony Integration Task in Aging
Source: Front Aging Neurosci. 2018 Oct 9;10:316. doi: 10.3389/fnagi.2018.00316 (PMC6189604; doi:10.3389/fnagi.2018.00316)
Supplement: Supplementary file 1 [file Table_1.DOCX]

**Supplementary Material**

Table 1 Statistical results of mean PLI and network metrics for the different stimulus conditions in the theta band.

| stimuli | MPLI | Eg | Eloc | Degree |
| --- | --- | --- | --- | --- |
| A-V | t=-0.078,p=0.938 | t=0.148,p=0.883 | t=-0.830,p=0.410 | t=-0.078,p=0.938 |
| A-AV | **t=-7.052,p=0.000** | **t=-7.403,p=0.000** | **t=-10.106,p=0.000** | **t=-7.591,p=0.000** |
| A-A50V | t=0.304,p=0.763 | t=0.150,p=0.809 | t=-0.727,p=0.471 | t=0.875,p=0.386 |
| A-A100V | **t=-5.581,p=0.000** | **t=-6.716,0.000** | **t=-7.062,p=0.000** | **t=-6.402,p=0.000** |
| A-V50A | **t=-9.409,p=0.000** | **t=-9.556,p=0.000** | **t=-7.281,p=0.000** | **t=-9.716,p=0.000** |
| A-V100A | **t=-3.863,p=0.005** | **t=-3.602,p=0.008** | **t=-3.814,p=0.001** | **t=-4.037,p=0.002** |
| V-AV | **t=-8.419,p=0.000** | **t=-9.305,p=0.000** | **t=-10.028,p=0.000** | **t=-9.725,p=0.000** |
| V-A50V | t=0.261,p=0.795 | t=1.090,p=0.281 | t=0.185,p=0.854 | t=0.569,p=0.572 |
| V- A100V | **t=-5.231,p=0.000** | **t=-4.379,p=0.000** | **t=-4.554,p=0.001** | **t=-4.902,p=0.000** |
| V-V50A | **t=-8.471,p=0.000** | **t=-8.853,p=0.000** | **t=-6.767,p=0.000** | **t=-9.341,p=0.000** |
| V-V100A | t=-2.123,p=0.686 | t=-1.865,p=0.068 | t=-1.448,p=0.231 | t=-1.056,p=0.284 |
| AV-A50V | **t=6.817,p=0.000** | **t=8.186,p=0.000** | **t=8.021,p=0.000** | **t=7.568,p=0.000** |
| AV-A100V | **t=3.743,p=0.009** | **t=4.477,p=0.000** | t=1.858,p=0.089 | **t=4.154,p=0.002** |
| AV-V50A | t=-0.620,p=0.538 | t=-1.778,p=0.081 | t=-0.235,p=0.815 | t=-0.934,p=0.355 |
| AV-V100A | **t=5.992,p=0.000** | **t=5.111,p=0.000** | **t=5.447,p=0.000** | **t=6.165,p=0.000** |
| V50A-A50V | **t=9.023,p=0.000** | **t=9.720,p=0.000** | **t=6.460,p=0.000** | **t=9.522,p=0.000** |
| V50A-A100V | **t=4.825,p=0.000** | **t=5.911,p=0.000** | t=1.677,p=0.100 | **t=5.464,p=0.000** |
| V50A-V100A | **t=9.543,p=0.000** | **t=7.622,p=0.000** | **t=5.016,p=0.000** | **t=9.373,p=0.000** |
| A50V-A100V | **t=-5.950,p=0.000** | **t=-7.494,p=0.000** | **t=-6.383,p=0.000** | **t=-6.155,p=0.000** |
| A50V-V100A | **t=-3.655,p=0.010** | **t=-4.830,p=0.000** | t=-2.064,p=0.094 | **t=-4.161,p=0.002** |
| A100V-V100A | **t=3.726,p=0.011** | t=1.841,p**=**0.152 | **t=3.503,p=0.024** | **t=3.510,p=0.023** |

The significant effects (P < 0.05) were indicated by bold letter.
