# Supplementary material for: Increased Functional Brain Network Efficiency During Audiovisual Temporal Asynchrony Integration Task in Aging
Source: Front Aging Neurosci. 2018 Oct 9;10:316. doi: 10.3389/fnagi.2018.00316 (PMC6189604; doi:10.3389/fnagi.2018.00316)
Supplement: Supplementary file 2 [file Table_2.DOCX]

**Supplementary Material**

Table 2 Statistical results of mean PLI and network metrics for the different stimulus conditions in the alpha band.

| stimuli | MPLI | Eg | Eloc | Degree |
| --- | --- | --- | --- | --- |
| A-V | t=0.579,p=0.565 | t=0.940,p=0.352 | t=0.430,p=0.669 | t=1.011,p=0.317 |
| A-AV | **t=-3.222,p=0.019** | t=-1.975,p=0.686 | t=-1.435,p=0.157 | t=-2.321,p=0.311 |
| A-A50V | **t=-4.624,p=0.000** | **t=-3.661,p=0.012** | t=-1.478,p=0.145 | **t=-3.990,p=0.003** |
| A-A100V | **t=-3.248,p=0.049** | t=-1.796,p=0.078 | t=-1.235,p=0.223 | t=-2.429,p=0.424 |
| A-V50A | **t=-6.255,p=0.000** | **t=-5.709,p=0.000** | **t=-4.768,p=0.000** | **t=-5.999,p=0.000** |
| A-V100A | t=-1.748,p=0.086 | t=-0.416,p=0.679 | t=-1.841,p=0.071 | t=-0.886,p=0.380 |
| V-AV | **t=-3.137,p=0.013** | t=-1.331,p=0.168 | t=-1.809,p=0.076 | t=-2.703,p=0.074 |
| V-A50V | **t=-3.881,p=0.002** | **t=-3.580,p=0.014** | t=-1.658,p=0.104 | **t=-3.781,p=0.004** |
| V- A100V | t=-1.942,p=0.090 | t=-1.213,p=0.659 | t=-1.503,p=0.139 | t=-2.657,p=0.198 |
| V-V50A | **t=-5.851,p=0.000** | **t=-5.853,p=0.000** | **t=-4.856,p=0.000** | **t=-6.166,p=0.000** |
| V-V100A | t=-2.898,p=0.100 | t=-1.563,p=0.124 | t=-2.727,p=0.181 | t=-2.455,p=0.330 |
| AV-A50V | t=-0.654,p=0.516 | t=-0.690,p=0.494 | t=-0.022,p=0.983 | t=-1.077,p=0.286 |
| AV-A100V | t=1.030,p=0.308 | t=0.860,p=0.394 | t=0.305,p=0.762 | t=0.666,p=0.508 |
| AV-V50A | **t=-3.401,p=0.030** | **t=-4.342,p=0.002** | **t=-3.599,p=0.018** | **t=-4.311,p=0.002** |
| AV-V100A | t=1.477,p=0.146 | t=1.384,p=0.172 | t=0.552,p=0.583 | t=1.229,p=0.225 |
| V50A-A50V | **t=3.128,p=0.021** | **t=3.770,p=0.007** | **t=4.622,p=0.001** | **t=3.682,p=0.013** |
| V50A-A100V | **t=3.801,p=0.006** | **t=4.695,p=0.000** | **t=3.872,p=0.007** | **t=4.260,p=0.001** |
| V50A-V100A | **t=4.208,p=0.002** | **t=4.619,p=0.000** | t=3.027,p=0.088 | **t=4.552,p=0.001** |
| A50V-A100V | t=1.791,p=0.079 | t=1.903,p=0.063 | t=0.311,p=0.757 | t=1.874,p=0.467 |
| A50V-V100A | t=1.160,p=0.577 | t=1.240,p=0.553 | t=-0.443,p=0.659 | t=2.205,p=0.557 |
| A100V-V100A | t=0.748,p=0.458 | t=0.921,p=0.362 | t=-0.861,p=0.393 | t=0.810,p=0.422 |

The significant effects (P < 0.05) were indicated by bold letter.
