# Supplementary material for: Increased Functional Brain Network Efficiency During Audiovisual Temporal Asynchrony Integration Task in Aging
Source: Front Aging Neurosci. 2018 Oct 9;10:316. doi: 10.3389/fnagi.2018.00316 (PMC6189604; doi:10.3389/fnagi.2018.00316)
Supplement: Supplementary file 3 [file Data_Sheet_1.PDF]

Supplementary Material

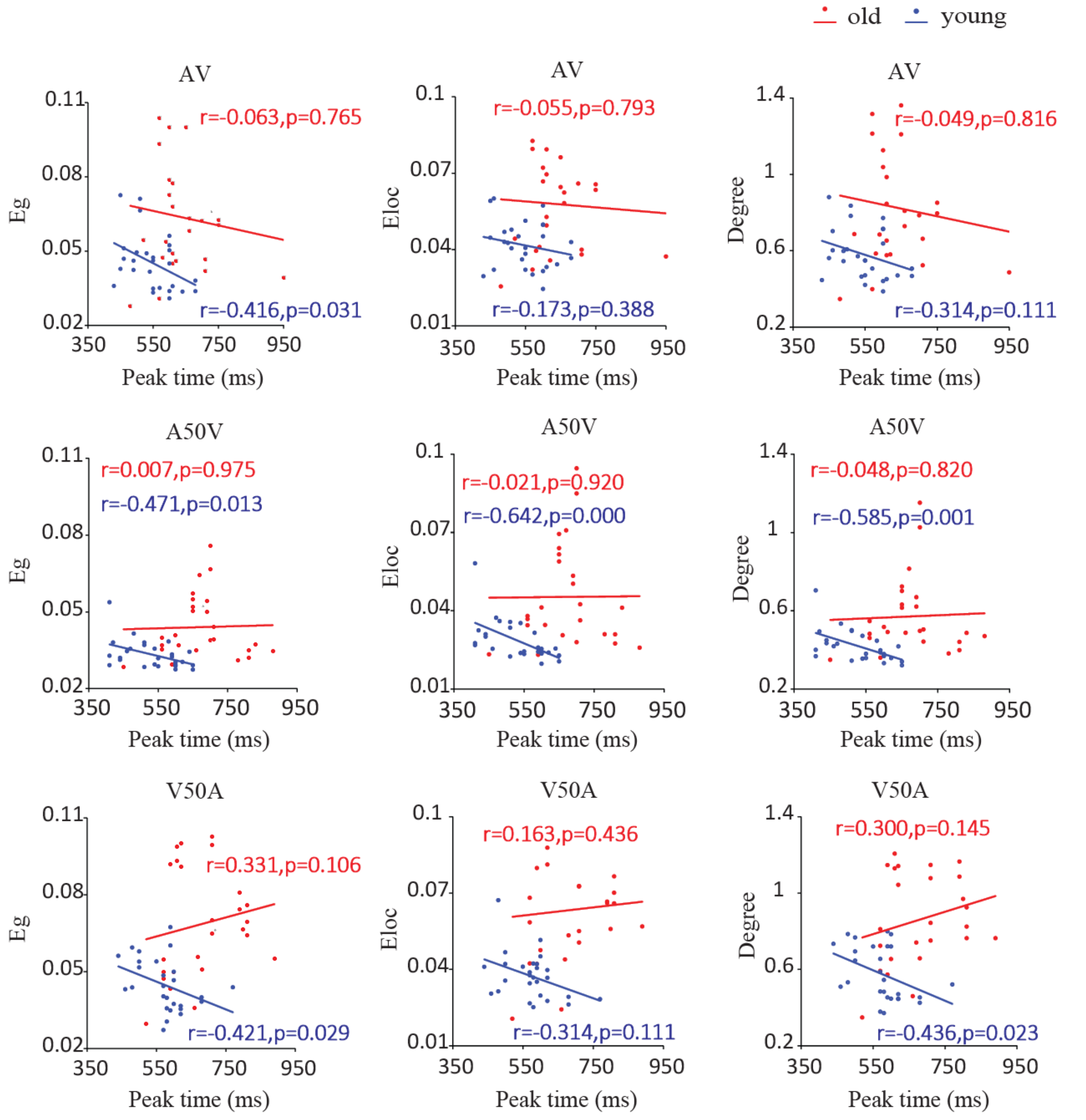

Figure 1. In the theta band, spearman correlations between the network metrics and the peak time in conditions with significant correlations. Global efficiency (Eg), local efficiency (Eloc), and degree have significant negative correlations with the peak time for young adults during AV, A50V, V50A conditions.

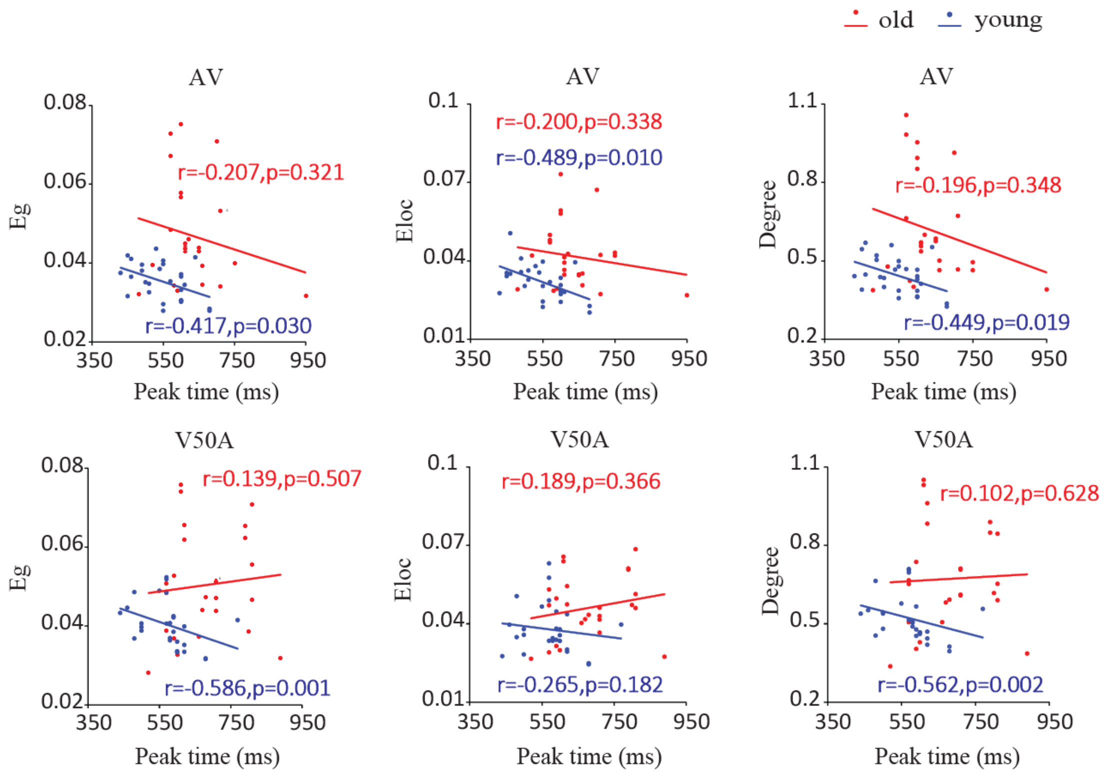

Figure 2. In the alpha band, spearman correlations between the network metrics and the peak time in conditions with significant correlations. Global efficiency (Eg), local efficiency (Eloc), and degree have significant negative correlations with the peak time for young adults during AV and V50A conditions.
